# Introduction to the Special Issue on “State-of-the-Art Sensor Technology in Japan 2015”

**DOI:** 10.3390/s16091350

**Published:** 2016-08-23

**Authors:** Masahiro Tokumitsu, Yoshiteru Ishida

**Affiliations:** 1 Department of Electrical and Control Engineering, National Institute of Technology, Yonago College, 4448 Hikonacho, Yonago 683-0854, Tottori, Japan; tokumitsu@yonago-k.ac.jp; 2 Department of Computer Science and Engineering, Toyohashi University of Technology, 1-1 Hibarigaoka, Tempaku-cho, Toyohashi 441-8580, Aichi, Japan

This Special Issue, “State-of-the-Art Sensor Technology in Japan 2015”, collected papers on different kinds of sensing technology: fundamental technology for intelligent sensors, information processing for monitoring humans, and information processing for adaptive and survivable sensor systems. This series published former two issues on state-of-the-art sensor technology in Japan in 2010 [1] and 2012 [2]. We selected the same aim in the same direction as in the previous ones: the monitoring of humans and the environment. However, the details of the topics have advanced from the former two issues. This issue covered fundamental achievements for intelligent sensors, information processing techniques for monitoring humans and children, and information processing for adaptive and survivable sensor systems.

## 1. Deepening of Sensing Technologies: Sensors and Information Processing

Japan faces two critical issues as humans and environmental issues. This Special Issue also selected the same topics as in the series of “State-of-the-Art Sensor Technology in Japan” in 2010 and 2012. However, Japan’s demographics are still rapidly changing as the population ages. The number of older people is growing, while the number of children is decreasing due to the falling birthrate. A safe and secure society for humans and children are required to solve these issues.

On the other hand, the environmental issues are also varied in Japan. Japan faced earthquakes, the 2016 Kumamoto earthquakes, on 16 April in 2016. The Kumamoto earthquakes also damaged more than 10 buildings of the Kumamoto Castle, which are nationally designated as important cultural properties. Not only that, but the earthquakes also damaged social infrastructures such as old bridges, roads, and mountains; therefore, they should be monitored and repaired if necessary. Thus, sensing technology is a key for a safe and secure society.

After the Greatest East Japan Earthquake on 11 March 2011, the resilience of information systems has been an important issue. Various kinds of resilient systems have been proposed and investigated extensively. Information and Communication Technology (ICT) is a fundamental technology for resilient systems. Recently, the Internet of Things (IoT) is also rapidly spreading and being used in society. Wearable devices are also available (e.g., smart watches, computer glasses, and wearable health monitors). Image sensors (cameras) are placed at roads and buildings anywhere, and they are operational. Thus, various kinds of data can be collected from these sensors of this sort. The next step is to construct survivable sensor systems that run even under even disaster situations while gathering data. Information processing techniques would play major roles in achieving the durable survivability of the resilient systems.

## 2. Fundamental Technology for Intelligent Sensors

Fundamental studies on sensors themselves are critical to develop intelligent sensors. Studies on the different kinds of sensors have been reported in this Special Issue, including X-ray sensors, infrared sensors, and biosensors. Furthermore, the development process and cost also should be considered to spread the use of the new sensors. We included the following five studies on fundamental research for the development of the sensors.

Matsuura et al. [3] investigated low-cost X-ray detectors with gated silicon drift detectors (GSDDSs). The X-ray detectors are required for on-site inspections of traces of hazardous elements in food and soil. The thickness of GSDDs is a key parameter for developing low-cost X-ray detectors. The study reported that the simulation results showed improvement of GSDDs formed from Si wafers. 

Takagawa et al. [4] investigated the detection wavelength control of uncooled infrared sensors by using two-dimensional lattice plasmonic absorbers. Wavelength-selective uncooled infrared (IR) sensors can be used for various kinds of applications such as fire detection, gas analysis, and biomedical analysis. For uncooled IR sensors, the study investigated the detailed effects of the lattice structures on the detection wavelength. The authors compared the square lattice with the triangular one and reported their design flexibility.

Yoshihara et al. [5] investigated new ratiometric molecular probes RP1 and RP2 for sensing oxygen levels in cells. Molecular oxygen is critical in cell metabolism and is also a key substrate in energy generation in aerobic organisms. The detection of oxygen levels in living cells is of great importance not only in cell biology, but also in other fields such as physiology and pathophysiology. The study reported that the proposed radiometric oxygen sensors exhibited improved cellular uptake efficiencies, and the radiometric images of living cells responded clearly to the oxygen concentration of the cell incubator.

Harada et al. [6] developed a taste sensor using lipid-polymer membranes to evaluate the taste of foods, beverages, and medicines. The response of the taste sensor, measured as a change in the membrane potential caused by adsorption (CPA), corresponds to the aftertaste felt by humans. The study investigated the relationship between the CPA value and epigallocatechin gallate (EGCg). The results of this study would contribute to the sensitivity of the taste sensors.

Camou [7] investigated a new technique for noninvasive and continuous monitoring of blood glucose levels. Monitoring blood glucose levels (BGL) is important for human health. BGL is a key quantity for monitoring human health because BGL is a critical parameter related to diabetes. There are several sensors commercially available to monitor BGL. The proposed technique is photoacoustic-based and noninvasive while commercial sensors are invasive with finger-pricking. The author reported that the proposed technique would reduce the patient’s burden for inspecting BGL.

## 3. Sensing Technology for Human Monitoring

Japan faces both human issues and environmental issues. For human issues, Japan’s demographics are rapidly changing as the population ages. Further, the falling birthrate also is quickly decreasing. Both issues (older people and the falling birthrate) are urgent matters to solve. A safe and secure society is an important environment for older people and children. Three studies are introduced in this Special Issue on monitoring human and children.

Hashizaki et al. [8] studied weekly sleep pattern variations at home with a contactless biomotion sensor. The sleep of people is restricted or disturbed by social obligations such as work. Sleep plays a major role in maintaining a healthy lifestyle. The authors recorded the sleep pattern of people using a contactless biomotion sensor with radiofrequency waves. The study reported that sleep of younger people indicated sleep delays on both weekdays and on the weekend due to social obligations.

Kaneko et al. [9,10] studied a measurement system for soft neurological signs (SNS) using acceleration and angular velocity sensors. They applied the system and method for children with Attention Deficit Hyperactivity Disorder (ADHD). Soft neurological signs are evident in the motor performance of children and disappear as the child grows up. The data observed by the proposed measurement system would quantify age-appropriate developmental change SNS for children. Furthermore, the authors considered the data of the motor performance of children with ADHD. The statistical analysis of the data revealed that children with ADHD showed a lag of several years behind typically developing children.

## 4. Intelligent Information Processing for Adaptive and Survivable Sensor Systems

The adaptiveness and resilience of sensor systems are still critical issues. The resilience of sensor systems has been studied widely and extensively since the accident of the Greatest East Japan Earthquake on 11 March 2011. However, Japan faced the similar earthquakes, the Kumamoto earthquakes, on 16 April in 2016. The Kumamoto earthquakes damaged the daily life of citizens, buildings, houses, and even important cultural properties of Kumamoto Castle. Sensing technologies for saving humans and recovering daily life are critical issues. Especially the resilience of the sensor systems is a key technology for natural disasters.

Miyazaki et al. [11] proposed an adaptive algorithm for multiple human tracking using binary infrared sensors. The proposed algorithm is able to estimate multiple human movement paths in a room without a priori knowledge of the number of humans. Furthermore, the proposed algorithm uses simple binary infrared sensors to construct the sensor system. This point is valuable as an application in homes. The study finally reported that the proposed algorithm showed adaptability, and it could handle the changes of the number of humans in the room.

Tokumitsu et al. [12] proposed a framework for the resilient sensor networks. The sensor systems are constructed by a huge amount of sensors and they communicate with each other to exchange messages. The integration framework is necessary to build the aggregate system of the sensors. The proposed framework involves resilience against missing sensors. The data of the missing sensors are interpolated by spatiotemporal interpolation with predefined profiles among sensors. The proposed framework would help in the management of large-scale sensor networks since the missing sensors are replaced with virtual sensors.

## 5. Toward the Next Generation of Sensing Technology

This Special Issue introduced various kinds of research on fundamental studies of sensors and information processing techniques. Investigation of a sensor itself is a critical issue. Further, the integration of the sensors is also an important issue to construct adaptive and survivable sensor systems. The integration of the sensors could detect the anomaly of the system itself or of monitoring targets. Information processing creates higher-level sensors (virtual sensors) to replace faulty sensors or expands the sensor system itself. The collective sensors [13] would produce valuable information by communication and processing data. Intelligent information processing and network technology are also key parts of sensing technologies. These three technologies, sensors, intelligent information processing, and the network, would enhance safe and secure society for humans and the environment.
